# A mysterious cloak: the peptidoglycan layer of algal and plant plastids

**DOI:** 10.1007/s00709-023-01886-y

**Published:** 2023-08-21

**Authors:** Alexander I. MacLeod, Michael R. Knopp, Sven B. Gould

**Affiliations:** Institute for Molecular Evolution, Heinrich Heine University of Düsseldorf, 40225 Düsseldorf, Germany

**Keywords:** Plastid evolution, Peptidoglycan, Murein layer, Plant evolution, Chloroplastida

## Abstract

The plastids of algae and plants originated on a single occasion from an endosymbiotic cyanobacterium at least a billion years ago. Despite the divergent evolution that characterizes the plastids of different lineages, many traits such as membrane organization and means of fission are universal—they pay tribute to the cyanobacterial origin of the organelle. For one such trait, the peptidoglycan (PG) layer, the situation is more complicated. Our view on its distribution keeps on changing and little is known regarding its molecular relevance, especially for land plants. Here, we investigate the extent of PG presence across the Chloroplastida using a phylogenomic approach. Our data support the view of a PG layer being present in the last common ancestor of land plants and its remarkable conservation across bryophytes that are otherwise characterized by gene loss. In embryophytes, the occurrence of the PG layer biosynthetic toolkit becomes patchier and the availability of novel genome data questions previous predictions regarding a functional coevolution of the PG layer and the plastid division machinery-associated gene FtsZ3. Furthermore, our data confirm the presence of penicillin-binding protein (PBP) orthologs in seed plants, which were previously thought to be absent from this clade. The 5-7 nm thick, and seemingly unchanged, PG layer armoring the plastids of glaucophyte algae might still provide the original function of structural support, but the same can likely not be said about the only recently identified PG layer of bryophyte and tracheophyte plastids. There are several issues to be explored regarding the composition, exact function, and biosynthesis of the PG layer in land plants. These issues arise from the fact that land plants seemingly lack certain genes that are believed to be crucial for PG layer production, even though they probably synthesize a PG layer.

## Introduction

Depending on the dating method, plastids emerged between 1.2 and 2.5 billion years ago (Bowles et al. 2023). Part of transforming an endosymbiont into an organelle involves transferring the majority of genetic information to the nucleus of the host cell through endosymbiotic gene transfer (EGT) (Deusch et al. 2008). Hence, the vast majority of photosynthesis- and plastid biogenesis-associated genes are cytosolically translated and then imported (Miyagishima et al. 2014; Knopp et al. 2020; Dowson et al. 2022). Enzymes of peptidoglycan (PG) layer biosynthesis, if present, are no exception. The peptidoglycan polymer provides bacterial cell walls with a structure and rigidity to protect themselves against biotic and abiotic stressors such as osmotic pressure, bacteriophages, heat, and salinity (Vollmer et al. 2008). The PG layer biosynthetic toolkit of the Chloroplastida is made up of ten key proteins: seven Mur enzymes (MurA-MurG), a DDL ligase (d-alanine:d-alanine ligase), the MraY enzyme, and penicillin-binding proteins (PBPs) (Dowson et al. 2022). PBPs have hitherto only been identified in algae, bryophytes, and seedless vascular plants (van Baren et al. 2016).

The presence of a PG layer in the plastids of the Archaeplastida has been documented for the Glaucophyta and Chloroplastida, but not yet for the Rhodophyta (Björn 2020). A cyanelle, the glaucophyte plastid, possesses a reduced yet still relatively thick PG layer between its two membranes with consequences for protein import (Steiner et al. 2005). The PG layer is also present in some members of the green lineage, such as mosses, but the degree to which this trait is conserved in multiple clades remains unresolved (Bachy et al. 2022). And while the peptidoglycan layer of mosses has been functionally characterized in parts (Hirano et al. 2016; Dowson et al. 2022), the same cannot be said for other embryophytes that possess orthologs for a full PG biosynthetic toolkit.

Understanding how the PG layer has evolved within the green lineage can provide valuable insights into the evolution of various aspects of plastid development, such as the timing of specific gene losses and gains. This could potentially clarify why some organisms within the Chloroplastida have unique plastid characteristics (Hirano et al. 2016; Li et al. 2017; de Vries and Gould 2018; MacLeod et al. 2022). In this study, we undertake a comprehensive and evidence-based phylogenomic approach on 48 genomes from the Chloroplastida supergroup and aim to delineate the phylogenetic distribution and evolution of the PG layer in the green lineage. We highlight that genes encoding proteins associated with PG layer biosynthesis have an uncommon phylogenetic distribution in the Chloroplastida and that the PG layer did not evolve concurrently with a component of the plastid division machinery, FtsZ3, as recently suggested (Grosche and Rensing 2017). The results underscore the PG layer’s existence in gymnosperms and spermatophytes, for which dedicated studies exploring its biological relevance for the plastid organelle are surprisingly sparse.

## Material and methods

### Determining the phylogenetic distribution of PG layer biosynthetic enzymes in the Chloroplastida, and plastid division components in chlorophyte algae

Protein sequence IDs of the ten key enzymes involved in peptidoglycan layer biosynthesis were retrieved from the genome of the liverwort *Marchantia polymorpha* and used as queries for the identification of orthology clusters (Bowman et al. 2017). OrthoFinder version 2.5.4 was used to identify orthologs among the input genomes from 48 Chloroplastida members (Goodstein et al. 2012; Hori et al. 2014; O’Leary et al. 2016; Bowman et al. 2017; Li et al. 2018, 2020b, 2020a ; Nishiyama et al. 2018; Cheng et al. 2019; Wang et al. 2020; Zhang et al. 2020; Grigoriev et al. 2021; Huang et al. 2022), with a BLASTp e-value threshold of 1 × 10^−9^. The phylogenetic distribution of enzymes involved in peptidoglycan layer biosynthesis was determined by examining the presence or absence of orthologous groups (orthogroups) containing these proteins across different members of the Chloroplastida. This exact pipeline was replicated to determine the presence/absence of plastid division components in 37 chlorophyte algae and one Prasinodermophyta (Li et al. 2020b; Grigoriev et al. 2021). Furthermore, where a given orthology cluster contained a protein family, which was the case for FtsZ proteins, phylogenetic trees were constructed to separate each protein into a respective subfamily. Sequence alignments were undertaken using MAFFT v7.471 using the LINSI parameter, with tree building being undertaken using IQ-TREE v2.0.3 using an automated selection model, with a 100 non-parametric bootstraps (Katoh et al. 2002; Minh et al. 2020). Finally, we used the SHOOT.bio phylogenetic application to determine whether PG layer biosynthetic genes in some seed plants branch within the terrestrial clade (Emms and Kelly 2022).

### Phylogenetic species tree construction

The species tree is based on a weighted concatenated alignment from 11 individual alignments. The first step was to calculate protein families including all sequences from the 48 analyzed genomes. Pairwise local identities were determined via DIAMOND (v2.0.1) and filtered for all reciprocal best blast hit pairs with at least 40% local sequence identity and a maximum e-value of 1 × 10^−10^ (Buchfink et al. 2015). A total of 210 protein clusters contained sequences from all 48 genomes; however, no single-copy gene cluster was found. To create a robust reference tree, 11 protein families were chosen in which only few genomes were represented by more than one sequence. For these clusters, alignments were calculated with MAFFT v7.471 using the LINSI parameter and the duplicate sequences were manually removed, favoring the copies that did not show major deletions or insertions to yield a robust phylogeny (Katoh et al. 2002). All 11 alignments were concatenated while equalizing their phylogenetic signal using a weighted concatenation approach. The final tree was built by IQ-TREE v2.0.3 (Minh et al. 2020) with 100 non-parametric bootstraps using the LG + F + R7 substitution model. Best-fit model identification was via IQ-TREE’s model finder (Minh et al. 2020). Tree trimming and visualization was carried out using the ggtree R package (Yu et al. 2017). A species tree of chlorophytes—used to plot the phylogenetic distribution of plastid division machinery components in this phylum—was estimated using STAG in the OrthoFinder run (Emms and Kelly 2015, 2015, 2018).

### Delineating domain architecture and function of orthologous sequences

InterProScan v5 (Jones et al. 2014) was used to delineate the basic domain architecture and function of protein sequences involved in peptidoglycan layer biosynthesis. The program was used to identify protein domains, annotate their functions, and determine the arrangement and composition of the domains in the protein sequences.

## Results

### Structural conservation of PG layer biosynthetic enzymes across the Chloroplastida

**Fig. 1:**
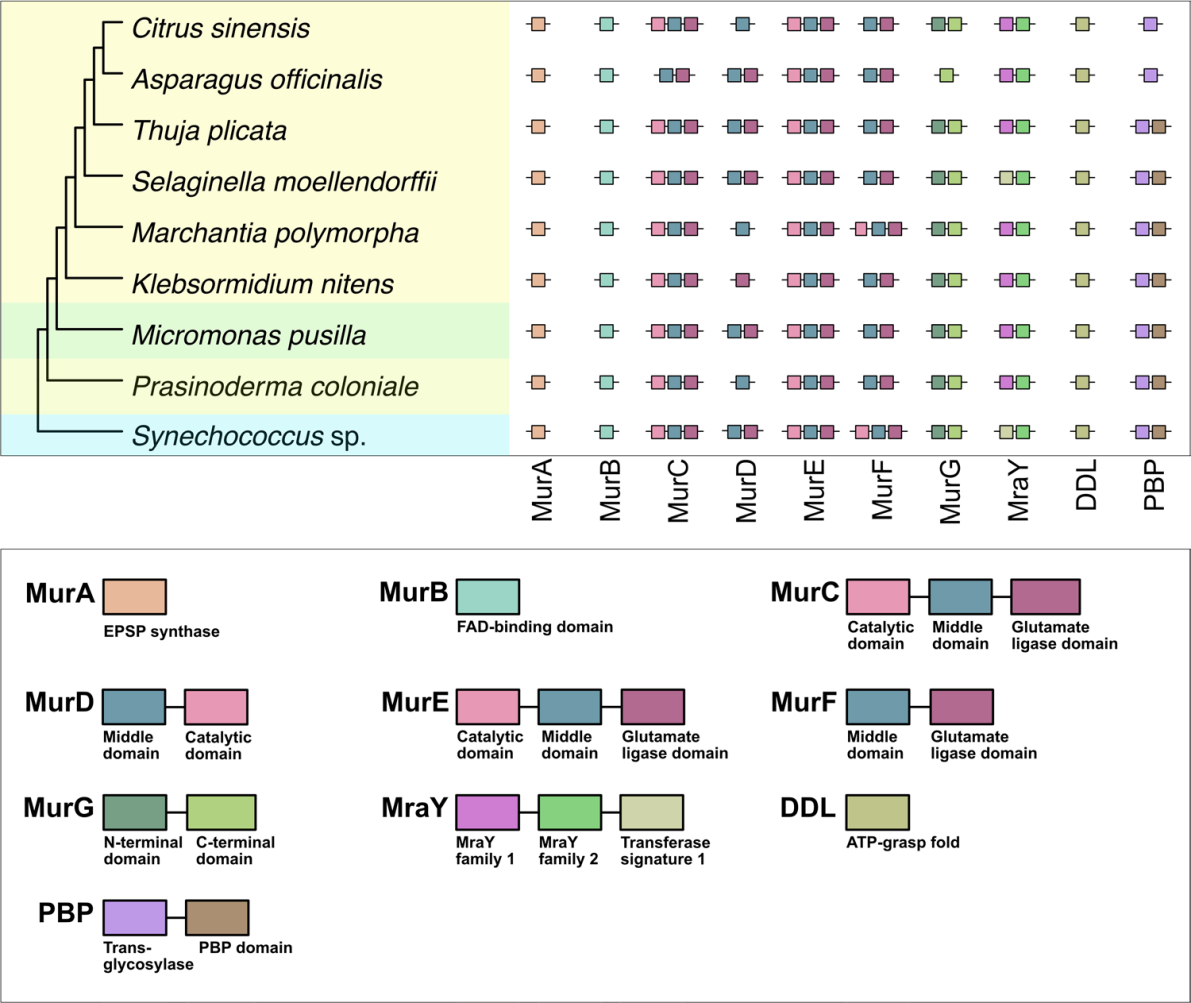
The PG layer biosynthetic toolkit is structurally well conserved from cyanobacteria to angiosperms. Cy*, Cyanobacteria; P*, Prasinodermophyta; Chl*, Chlorophyta

Protein domain and gene ontology analyses show a high level of structural conservation in PG layer biosynthetic enzymes, from algae to angiosperms, and confirm that these proteins likely play key roles in peptidoglycan biosynthesis in the species analyzed (Fig. 1). Furthermore, while previous studies have suggested that the PG layer was differentially lost in the MRCA of seed plants (spermatophytes) (Grosche and Rensing 2017), the full toolkit for the biosynthesis of peptidoglycan is identified in at least three phylogenetically distant members of the seed clade: *Thuja plicata* (Gymnosperms), *Asparagus officinalis* (Monocots), and *Citrus sinensis* (Eudicots) (Fig. 1). This includes the identification of PBP family orthologs in the seed clade.

### No evident correlation between the presence of the PG layer and any of the three FtsZ proteins

**Fig. 2:**
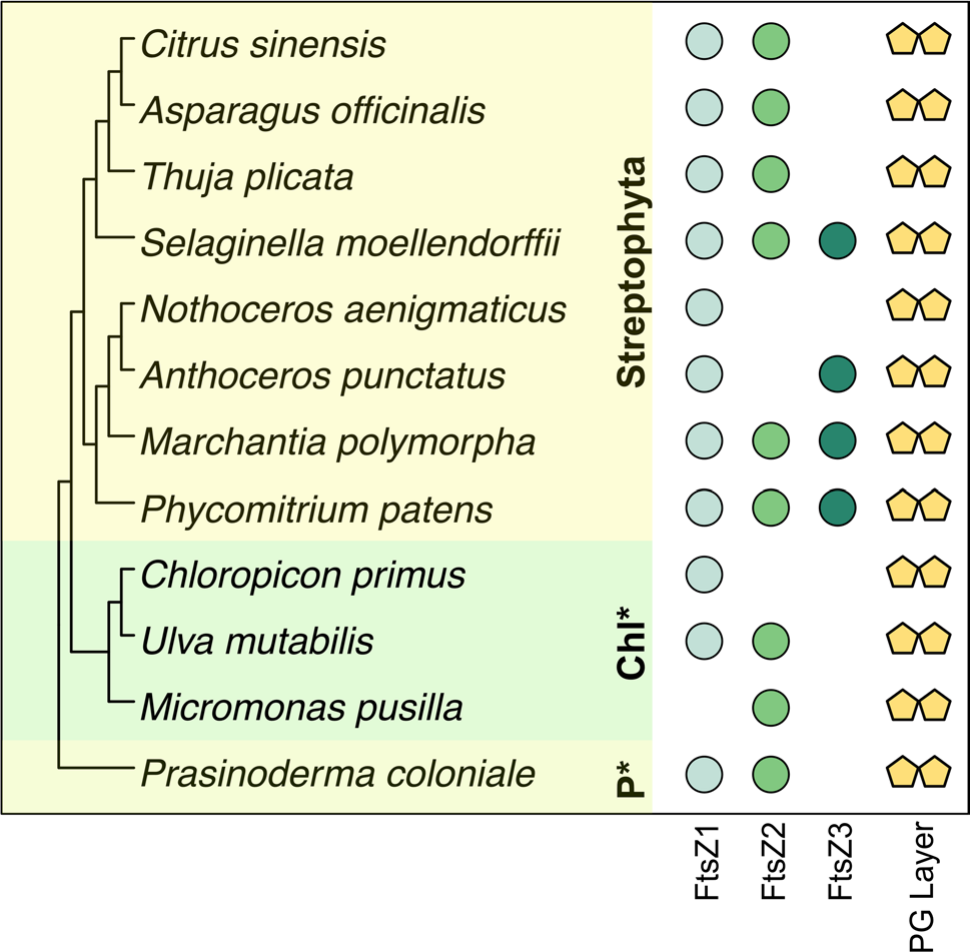
Phylogenetic distribution of the FtsZ plastid division proteins in Chloroplastida that likely have a PG layer, highlighting the unlikely coevolution between any specific FtsZ protein and the plastid enveloping murein layer. P*, Prasinodermophyta; Chl*, Chlorophyta. Ortholog metadata for FtsZs in the Streptophyta was obtained from MacLeod et al. (2022)

The PG layer plays a key role in regulating chloroplast division in bryophytes and streptophyte algae (Machida et al. 2006; Homi et al. 2009; Hirano et al. 2016; Grosche and Rensing 2017; Dowson et al. 2022). The GTPases FtsZ1, FtsZ2, and FtsZ3 are central to plastid division, and form versatile heteropolymers that establish constriction sites, facilitating the division of plastids in a coordinated and efficient way (Martin et al. 2009; Yoshida et al. 2016). FtsZ3 was suggested to play a role in regulating the biogenesis of the PG layer due to an alleged correlation between these two traits (Grosche and Rensing 2017). There are, however, multiple exceptions to this correlation. For example, the spermatophytes *Thuja plicata*, *Asparagus officinalis*, and *Citrus sinensis* possess orthologs representing a full enzymatic toolkit for PG layer biosynthesis (Fig. 2). In addition, the hornwort *Nothoceros aenigmaticus* and the phylogenetically distant chlorophytes, *Chloropicon primus*, *Ulva mutabilis*, and *Micromonas pusilla*, all likely possess a PG layer between their chloroplast membranes (Bachy et al. 2022; MacLeod et al. 2022), but lack FtsZ3 (Fig. 2). In summary, the now-available genomes do not support a PG layer and FtsZ3 coevolution or functional connection. In fact, it appears that the presence of the chloroplast PG layer is not dependent on the presence of any one specific protein of the FtsZ family (Fig. 2).

## Discussion

The unusual phylogenetic distributions of individual genes or even entire biosynthetic pathways are sometimes the result of the identification of bacterial false positive contaminations (Koutsovoulos et al. 2016; Husnik and McCutcheon 2018; Goig et al. 2020). This is unlikely to be the case regarding PG layer biosynthetic enzymes from *T. plicata*, *A. officinalis*, and *C. sinensis*, as they branch deep within the embryophyte clade (Emms and Kelly 2022). As such, all available data suggest a monophyletic origin of the pathway (Li et al. 2020b) and an independent differential loss in various taxa across the Archaeplastida (Bachy et al. 2022).

Most angiosperms do not seem to encode for a complete set of enzymes synthesizing the PG layer. However, they all share four enzymes related to the process (MurE, MurG, MraY, and DDL), called the “4-PGN” set, and recent experimental work suggests that two angiosperms, *Arabidopsis thaliana* and *Nicotiana benthamiana*, may have a PG layer surrounding their chloroplasts (Tran et al. 2023). If true, then it would suggest that these species use a different set of enzymes and biochemistry to synthesize parts of the PG layer, with the 4-PGN set playing a key role, therefore being retained. Intriguingly, the retention of the same set of genes (± 1) occurred independently in some chlorophyte algae such as *Micromonas commoda* (van Baren et al. 2016). It raises the question whether they have been retained for the same functional reason, which is likely but not proven.

While recent biochemical and metabolomic analyses suggest that components of the moss peptidoglycan biosynthetic pathway—specifically, the active sites of core ligase enzymes—display strict conservation in comparison to the PG layer biosynthetic pathway of cyanobacteria (Dowson et al. 2022), the FtsZ3 PDVM component is unlikely to play a role in PG layer biosynthesis in moss. Genome analyses, including our own, indicate that the PG layer exists in all three phyla of Chloroplastida (van Baren et al. 2016; Grosche and Rensing 2017; Li et al. 2020b). There is, however, no strict connection between FtsZ3, or any FtsZ gene, and the PG layer in terms of the FtsZ-based ring’s association with this cyanobacterial relic. Therefore, any gene from the FtsZ family can likely perform its role in regulating the formation of plastid division rings, indicating functional redundancy within this family.

## Conclusion

The peptidoglycan layer of chloroplasts was present in the MRCA of Chloroplastida and lost in most Chlorophyta and many Streptophyta, but retained in the Prasinodermophyta. Since the number of annotated genome assemblies for this latter phylum still stands at a mere one, it will be interesting for future genome mining experiments to elucidate whether the PG layer can be characterized—either biochemically or genomically—in this basal-branching green phylum. One can conclude that the PG layer is present in the chloroplasts of at least three phylogenetically distant spermatophytes, likely more, suggesting that peptidoglycan is more widespread in the chloroplasts of this phylum than previously thought. Moreover, the correlation between the presence of the PG layer and the plastid division protein FtsZ3 is no longer supported. Based on this evidence, upcoming studies should now focus on clarifying both the biochemical characteristics and the biological significance of the PG layer in angiosperms, which were previously thought to lack this ancient cyanobacterial feature.

## Data Availability

For questions about accessing the data from this study, please contact the corresponding author directly.
